# Titanium dioxide nanoparticles exaggerate respiratory syncytial virus-induced airway epithelial barrier dysfunction

**DOI:** 10.1152/ajplung.00104.2020

**Published:** 2020-07-08

**Authors:** Carrie C. Smallcombe, Terri J. Harford, Debra T. Linfield, Susana Lechuga, Vladimir Bokun, Giovanni Piedimonte, Fariba Rezaee

**Affiliations:** ^1^Department of Inflammation and Immunity, Lerner Research Institute, Cleveland Clinic Foundation, Cleveland, Ohio; ^2^Cleveland Clinic Lerner College of Medicine of Case Western Reserve University, Cleveland, Ohio; ^3^Tulane University School of Medicine, New Orleans, Louisiana; ^4^Centre for Pediatric Pulmonary Medicine, Cleveland Clinic Children’s, Cleveland, Ohio

**Keywords:** nanoparticles, reactive oxygen species, respiratory syncytial virus, tight junctions, titanium dioxide nanoparticles

## Abstract

Respiratory syncytial virus (RSV) is the leading cause of lower respiratory tract infections in children worldwide. While most develop a mild, self-limiting illness, some develop severe acute lower respiratory infection and persistent airway disease. Exposure to ambient particulate matter has been linked to asthma, bronchitis, and viral infection in multiple epidemiological studies. We hypothesized that coexposure to nanoparticles worsens RSV-induced airway epithelial barrier dysfunction. Bronchial epithelial cells were incubated with titanium dioxide nanoparticles (TiO_2_-NP) or a combination of TiO_2_-NP and RSV. Structure and function of epithelial cell barrier were analyzed. Viral titer and the role of reactive oxygen species (ROS) generation were evaluated. In vivo, mice were intranasally incubated with TiO_2_-NP, RSV, or a combination. Lungs and bronchoalveolar lavage (BAL) fluid were harvested for analysis of airway inflammation and apical junctional complex (AJC) disruption. RSV-induced AJC disruption was amplified by TiO_2_-NP. Nanoparticle exposure increased viral infection in epithelial cells. TiO_2_-NP induced generation of ROS, and pretreatment with antioxidant, *N*-acetylcysteine, reversed said barrier dysfunction. In vivo, RSV-induced injury and AJC disruption were augmented in the lungs of mice given TiO_2_-NP. Airway inflammation was exacerbated, as evidenced by increased white blood cell infiltration into the BAL, along with exaggeration of peribronchial inflammation and AJC disruption. These data demonstrate that TiO_2_-NP exposure exacerbates RSV-induced AJC dysfunction and increases inflammation by mechanisms involving generation of ROS. Further studies are required to determine whether NP exposure plays a role in the health disparities of asthma and other lung diseases, and why some children experience more severe airway disease with RSV infection.

## INTRODUCTION

Respiratory syncytial virus (RSV) is the most common cause of acute lower respiratory infection (ALRI) in young children worldwide (34, 40, 41, 76, 102). In 2015, more than 33 million occurrences of RSV-related ALRI have been estimated globally with 3 million children requiring hospitalization (74, 115). RSV is also a significant source of morbidity and mortality in elderly and high-risk adults (30, 75, 101). There are strong associations between RSV, persistent wheezing, and childhood asthma (47, 132). Despite extensive research, no effective treatment is available for RSV infection, aside from supportive care (101, 130). The virus mainly targets airway epithelial cells, triggering profound inflammation and diminishing integrity of the epithelial barrier by disrupting specialized intercellular structures termed apical junctional complexes (AJC) (98, 101). AJC comprise tight junctions (TJ) and adherens junctions (AJ) that are linked to the actin cytoskeleton (65, 85, 122). We and others have demonstrated that RSV stimulates increased permeability and a “leaky airway” both in vitro and in vivo (35, 99, 117).

Nearly all children become infected with RSV by age 2, and while most experience a mild, self-limiting illness, some develop severe ALRI and persistent airway disease (132). While many host factors associated with disease severity such as prematurity, age, or underlying chronic lung diseases are well-described (18, 76, 109), the associations of RSV infection with many environmental exposures are not yet fully understood. Exposure to ambient particulate matter (PM) has been linked to pulmonary diseases, such as asthma, bronchitis, otitis media, and severe viral infection in multiple epidemiologic studies (4, 6, 10, 12, 29, 37, 58, 70, 80a, 92, 108). Particulate matter comprises fine particles (FP) and ultrafine nanoparticles (NP), with aerodynamic diameters of <2.5 µm and <21 nm, respectively. As such, NP display a greater ability to be deposited and retained in the distal airways compared with coarser FP (87). Recent studies have shown that even short-term exposure to PM in a young age increases the risk of ALRI (39, 48). Inhaled NP have been demonstrated to become deposited in the lower airways (31, 95) and can translocate to the bloodstream and reach distant tissues and organs, including the brain (17, 50, 79, 80). This has been proposed to have a significant effect on asthma and allergies (90, 104). There is also increasing evidence to suggest that ingested NP alter barrier integrity to translocate through the gut and distribute throughout the body (15, 126).

Atmospheric nanoparticles are produced through manufacturing processes, motor vehicle exhaust emissions, or are derived from industrial and residential combustion processes (2). The rapid expansion of nanotechnology has seen a massive increase in engineered nanoparticle production (62, 68, 110). Currently, there are no standardized nanoparticle measurement methods and no federal standards for hazardous levels in the environment (8). A World Health Organization report revealed that toxic air is associated with more than one in four deaths among children younger than 5 yr old, and 93% of all children live in environments with air pollution levels above WHO guidelines (129). In 2016, ambient and household air pollution contributed to respiratory tract infections that resulted in the deaths of 543,000 children under 5 yr old globally. In addition, evidence suggests that even everyday household products produce aerosolized particulate matter (21).

Titanium dioxide (TiO_2_) nanoparticles are the most abundantly produced nanomaterial in commercial applications, present in many consumer products for use as an anticaking additive or whitening agent in paints, inks, cosmetics, toothpaste, and sunscreen (7, 88, 128). TiO_2_ particles have demonstrated the potential for triggering dose-dependent inflammation and injury of the lower airways (12, 22, 23, 28, 51, 56, 68, 78, 87, 106) and inducing endothelial leakiness (114). For example, inhalation has been shown to induce eosinophilic airway inflammation in rabbits (23) and enhance lung inflammation and airway hyperreactivity in murine models of asthma (51, 56) perhaps due to direct activation of lung dendritic cells (10). Exposure to NP also increased airway hyperresponsiveness and upregulation of neurogenic inflammation (113). Rat studies have suggested that treatment with TiO_2_-NP causes increased neutrophil infiltration into the airways and causes increased barrier permeability and cellular damage in lung tissue (52). TiO_2_-NP have also been shown to reduce the phagocytic activity of alveolar macrophages, potentially impeding clearance of particles and other pathogens from the lungs (97). Pertinently, TiO_2_-NP exposure increases susceptibility of epithelial cells to bacterial and viral infections (20, 134).

A workgroup report from the American Academy of Allergy, Asthma, and Immunology recently concluded that ambient particles should receive special attention for potential adverse health outcomes in humans (68). Specifically, there is a need to elucidate the interactions between NP exposure and viral infection especially during childhood, a critical period of vulnerability to environmental stimuli. We hypothesized that disruption of the epithelial barrier by nanoparticles worsens RSV-induced airway injury. We hypothesized that the coexposure to nanoparticles worsens RSV-induced airway epithelial barrier dysfunction. This study aimed to investigate the effect of short-term exposure to TiO_2_-NP on RSV-induced airway barrier disruption.

## MATERIALS AND METHODS

### 

#### Antibodies and other reagents.

The following monoclonal (mAbs) and polyclonal antibodies (pAbs) were used to detect tight junction and adherens junction proteins by immunofluorescent labeling and/or immunoblotting: anti-RSV A2 pAb, which reacts with glycoprotein G of the A2 strain (GTX70381; GeneTex, Irvine, CA), anti-β-catenin mAb, anti-GAPDH mAb (Abcam, Cambridge, MA), anti-zonula occludens (ZO)-1 pAb, anti-occludin mAb, anti-E-cadherin mAb (Thermo-Fisher, Waltham, MA), and cleaved caspase-3 pAb (Cell Signaling, Danvers, MA). Alexa Fluor 488- and 568=conjugated anti-rabbit and anti-mouse secondary antibodies were obtained from Thermo-Fisher Scientific (Waltham, MA). Mouse and rabbit secondary horseradish peroxidase-conjugated antibodies were purchased from GE Healthcare (Pittsburgh, PA). *N*-acetylcysteine (NAC) was purchased from Sigma.

#### Cell culture and RSV infection.

Immortalized human bronchial epithelial cells, 16HBE14o (16HBE), were provided by Dr. Dieter Gruenert (University of California, San Francisco). Cells were cultured in DMEM medium supplemented with 10% fetal bovine serum, HEPES, and penicillin-streptomycin antibiotics. To study epithelial barriers, cells were grown on collagen-coated Transwell-permeable supports (Corning, Tewksbury, MA) under liquid-liquid conditions. Primary human bronchial epithelial cells isolated from the lungs of pediatric donors were grown on Transwell membrane inserts under air-liquid interface (ALI) conditions as previously described (98). Transepithelial electric resistance (TEER) was evaluated using an EVOM2 voltohmmeter (World Precision Instruments, Sarasota, FL) and shown as % change from *t* = 0 as per previous studies (103). Experiments were performed when the polarized monolayers had a TEER reading >500 Ω x cm^2^, which occurred 7 days after plating for 16HBE and 6 wk for the primary cells (3–4 wk after maintaining primary cells in ALI).

#### Virus.

rrRSV [RSV derived from RSV A2 expressing the red fluorescent protein (RFP) upon replication] was a kind gift from Mark Peeples (Nationwide Children’s Hospital Research Institute, Columbus, OH) and Peter Collins (National Institute of Health, Bethesda, MD) (42, 98, 100, 117). Virus was propagated as previously described (98).

#### TiO_2_ particle preparation.

**Fig. 1.**
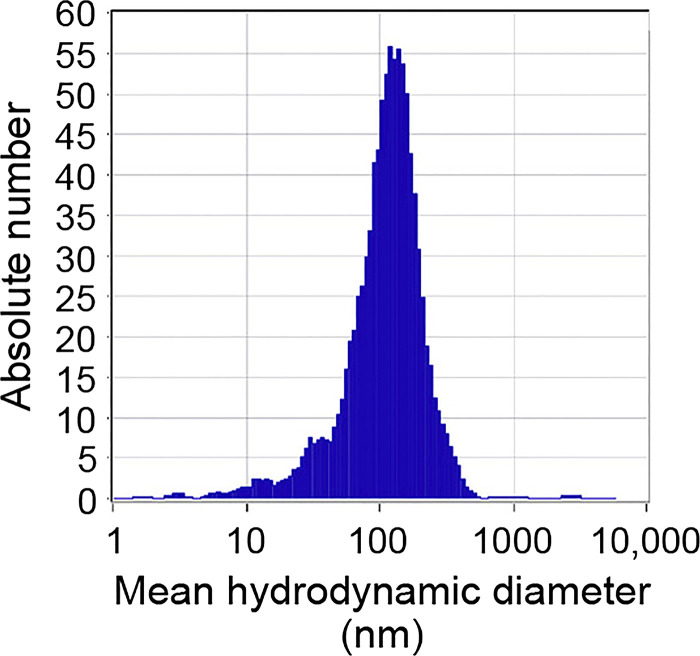
Characterization of titanium dioxide nanoparticles (TiO_2_-NP). Laser scattering microscopy was used to determine the size distribution in the fraction of TiO_2_-NP selected for cell studies. Graph shows that a narrow distribution of particle size peaked at a hydrodynamic diameter of 100 nm.

Ultrafine TiO_2_ nanoparticles (Sigma Aldrich, St. Louis, MO) were suspended in a biocompatible dispersion medium (DM) composed of 5.5 mM D-glucose, 0.6 mg/mL bovine serum albumin, and 0.01 mg/mL dipalmitoyl-phosphatidylcholine (DPPC) dissolved in sterile Ca^2+^- and Mg^2+^-free PBS as previously described (93). Solutions were sonicated for 10 min on ice immediately before use to avoid agglomeration. Agglomeration state of the particles in suspension was determined by dynamic light scattering and zeta potential analysis (Nicomp, Santa Barbara, CA), which yielded a mean hydrodynamic diameter of ~100 nm, and confirmed that the particles do not agglomerate in suspension (Fig. 1). Working concentrations of particle solution (10–100 µg/mL, equal to 10–100 µg/cm^2^) were achieved by addition of DM-particle suspensions directly to the apical cell surface.

#### Immunofluorescent labeling, confocal microscopy, and detection of fluorescently labeled RSV.

Epithelial cells cultured on Transwell membrane filters were fixed with cold methanol and subjected to immunofluorescence labeling as previously described (101, 117). Immunolabeled cell monolayers were examined by confocal microscopy using a Leica TCS-SP spectral laser-scanning confocal microscope. Epithelial cells infected with RFP-labeled RSV were visualized using a Lumen200 epifluorescence microscope (Prior, Cambridge, UK), and the number of RFP-positive cells was quantified. Additionally, fluorescent-focused unit (FFU) assay was performed to quantitatively analyze of RFP-positive cells by ImageJ (112).

#### Calcium switch assay.

To determine whether disruption of epithelial junctions affects viral replication, cells were incubated in low calcium (5 μM) culture medium in the presence or absence of RSV overnight as described (53, 65). Epithelial junctions are highly sensitive to calcium concentrations, and its removal and restoration from media can open and reseal AJCs (38). The next morning, wells were replaced with whole medium. On *day 2*, RFP-tagged virus in infected epithelial cells was visualized by fluorescence microscopy.

#### Epithelial permeability assays.

Transmonolayer flux of FITC-labeled dextran was measured as previously described (98, 101). Briefly, epithelial cells were cultured on Transwell membrane filters. Once confluent, FITC-labeled dextran (4 kDa; Sigma, St. Louis, MO) was dissolved in sterile PBS (to 540 μg/mL) and added to the apical chamber; medium in the basal chamber was replaced with sterile PBS. Samples were collected from the latter, and FITC intensity was recorded at an excitation wavelength of 485 nm and an emission wavelength of 528 nm using a FlexStation 3 (Molecular Devices, Sunnyvale, CA).

#### Measurement of reactive oxygen species.

Reactive oxygen species (ROS) generation was measured in subconfluent 16HBE cells using a fluorescent probe-based assay (Abcam, Cambridge, MA) as previously described (83, 89). Briefly, cells were preincubated with dichlorodihydrofluorescein diacetate (DCFDA) dye for 45 min, followed by exposure to TiO_2_-NP, RSV, their combination, or control cell medium (without phenol red) for the indicated times. Tert-butyl hydroperoxide mimics ROS activity to oxidize DCFDA to fluorescent DCF and was used as a positive control. Florescence intensity of the experimental samples was recorded at an excitation wavelength of 485 nm and an emission wavelength of 528 nm using a FlexStation 3.

#### Lactate dehydrogenase assay.

Lactate dehydrogenase (LDH) assay was performed as previously described (98, 103). Airway epithelial cells were exposed to RSV and/or TiO_2_-NP, and, after 48 h, cell culture medium was removed and centrifuged to remove cell debris. Supernatant of cells exposed to 2% Triton-X 100 was used as a positive control. Quantitation of LDH in cell culture supernatant was determined by commercially available LDH Cytotoxicity Detection kit (Takara Bio, Mountain View, CA).

#### Immunoblotting and Western blot analysis.

Confluent 16HBE cells were exposed to medium control, TiO_2_-NP, RSV, or a combination thereof. Cell lysates were collected with RIPA lysis buffer (with protease and phosphatase inhibitors) and centrifuged to remove cell debris. Western blot analysis of AJC proteins ZO-1, occludin, β-catenin, and E-cadherin was performed as previously described (117). Western blot analysis of cleaved caspase 3 was performed using 10 μL boiled cytochrome *c*-treated Jurkat cell lysate as a positive control. Viral protein expression in infected cells was assessed using anti-RSV A2 pAb, which reacts with glycoprotein G of the A2 strain (14). Images were taken using a MyECL imager (Thermo Scientific). The pixel density of each band was estimated with ImageJ software (National Institutes of Health, Bethesda, MD) and normalized to the lane-loading control, mouse anti-GAPDH (Abcam, Cambridge, UK). Results were expressed as a ratio of protein of interest to GAPDH and reported as the fold change from baseline. An uncropped Western blot for each antibody is shown in Supplemental Fig. S1 (see https://doi.org/10.6084/m9.figshare.12585704).

#### Extraction of RNA and quantitative RT-PCR analysis.

RSV Nucleocapsid (N) copy number was quantified as previously described (117). In short, RNeasy Mini Kit, automated on the Qiacube (Qiagen), was utilized to extract total RNA from lung tissues. Amplification and copy number determination of RSV N copy number were resolved using a commercially available Primerdesign Genesig Kit for Respiratory Syncytial Virus Type A (RSV-A) genomes from Primer Design (Oxford, UK). A lyophilized standard, when reconstituted, yields a quantified concentration of *N* copies per microliter. This is then used to quantify the number of *N* copies in the test samples.

#### Cytokine and chemokine analysis.

A human cytokine/chemokine magnetic bead panel (HCYTOMAG-60K-PX30; EMD-Millipore, Billerica, MA) was used to quantify the following known inflammatory cytokines and chemokines in 16HBE apical supernatant: interleukins IL-1ra, IL-1α, IL-1β, IL-2, IL-3, IL-4, IL-5, IL-6, IL-7, IL-8, IL-10, IL-12 (p40 and p70), IL-13, IL-15, IL-17, interferon-γ (IFNγ), IFNα2, CXCL-10 [IFNγ-induced protein 10 (IP-10)], tumor necrosis factor-α (TNFα), TNFβ, CCL-2 [monocyte chemoattractant protein (MCP)-1], CCL-3 [macrophage-inflammatory protein (MIP)-1α], CCL-4 (MIP-1β), CCL-5 (RANTES), CCL-11 (eotaxin), epidermal growth factor (EGF), granulocyte-colony stimulating factor (G-CSF), granulocyte-macrophage colony-stimulating factor (GM-CSF), and vascular endothelial growth factor (VEGF).

Similarly, a mouse cytokine/chemokine magnetic bead panel (MCYTOMAG-70K-PX32; EMD-Millipore, Billerica, MA) was used to quantify the following inflammatory cytokines and chemokines in mouse BAL: interleukins IL-1α, IL-1β, IL-2, IL-3, IL-4, IL-5, IL-6, IL-7, IL-9, IL-10, IL-12 (p40 and p70), IL-13, IL-15, IL-17, IFNγ, CXCL-10 (IP-10), TNFα, CXCL-1 [keratinocyte chemoattractant (KC)], leukemia inhibitory factor (LIF), CXCL5 (LIX), CCL-2 [monocyte chemoattractant protein (MCP)-1], CCL-3 [macrophage-inflammatory protein (MIP)-1α], CCL-4 (MIP-1β), CXCL-9 [monokine induced by IFNγ (MIG)], CXCL-2 (MIP-2), CCL-5 (RANTES), CCL-11(eotaxin), G-CSF, GM-CSF, macrophage-colony stimulating factor (M-CSF), and VEGF.

The standard, quality control, and samples were processed according to the manufacturer's instruction.

#### Animals.

Female C57BL/6 mice 6–8 wk old (average weight 18–20 mg) were purchased from Jackson Laboratories (Bar Harbor, ME) and intranasally instilled with 0.5–3 mg/kg (average 10–60 μg/mice) TiO_2_-NP suspended in a biocompatible dispersion medium (DM) as described above and/or RSV [9.6 × 10^6^ plaque-forming units (PFU)] according to our previous study (117). The TiO_2_-NP dose was according to recent studies that showed alveolitis and pulmonary inflammation in dosages above 30 μg/mice (94). Equal volumes of PBS or DM alone were administered as controls. Mice were euthanized on *day 4* post-RSV inoculation, corresponding with peak viral replication according to previous studies (117, 118). Lungs were harvested for analysis of inflammation: sections of formalin-fixed, paraffin-embedded lungs were stained with hematoxylin and eosin and examined by light microscopy; slides were blinded, and the extent of pathology involving bronchioles, peribronchioles, and perivascular tissue was evaluated as previously described (117). Bronchoalveolar lavage (BAL) was harvested for the quantification of white blood cell infiltration.

#### Ethics statement.

Human primary epithelial cells were isolated from human tissue from deceased pediatric donors. Tissue was provided by the International Institute for the Advancement of Medicine (IIAM) according to the procedures approved by the Cleveland Clinic. As such, the human tissue is exempt from requiring IRB approval as the use of this tissue is not considered as a human study by the Cleveland Clinic. All animal procedures used in this study adhered to the National Institutes of Health *Guide for the Care and Use of Laboratory Animals* and were reviewed and approved by the Institutional Animal Care and Use Committee (IACUC; approved protocol 2018-2030) of the Lerner Research Institute at the Cleveland Clinic. This facility is accredited by the Association for the Assessment and Accreditation of Laboratory Animal Care (AAALAC; accreditation number 000383) and is in compliance with federal law and NIH regulations.

#### Data analyses.

Data were analyzed using Prism software (GraphPad, San Diego, CA) and Microsoft Excel. Data are representative of three or more experiments and are presented as means and standard error. For comparison of two groups with parametric data, Student’s two-tailed *t*-test was used; for comparison of multiple groups, we performed one-way analysis of variance (ANOVA) followed by Dunnett’s post hoc test for all the groups of experiment. For the cytokine studies, we performed overall one-way ANOVA corrected for multiple cytokine testing using the Benjamini and Hochberg false discovery rate (FDR) method. For cytokines with FDR < 0.05, pairwise group comparisons were performed using Tukey or Dunnett multiple-comparison adjustments at overall significance levels of 0.05.

## RESULTS

### 

#### Titanium dioxide nanoparticles induce AJC disassembly and increase paracellular permeability in immortalized and primary human bronchial epithelial cells.

**Fig. 2.**
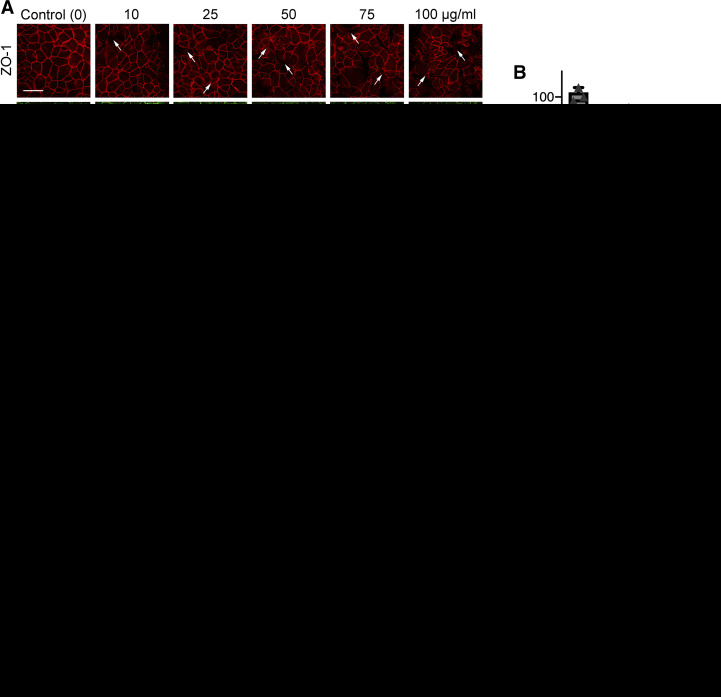
Titanium dioxide nanoparticles (TiO_2_-NP) induce disassembly of epithelial tight junctions. 16HBE cells were exposed to 10–100 μg/mL TiO_2_. Cells were immunofluorescently labeled for TiO_2_-NP apical junctional complex (AJC) proteins zonula occludens-1 (ZO-1), occludin, β-catenin, and E-cadherin (*A*). Arrows indicate areas of disruption. Permeability of the barrier was examined by transepithelial electrical resistance (TEER) at 48 h reading after exposure to TiO_2_-NP, and changes were normalized to the time zero for each group (*B*). Human primary epithelial cells were exposed to 10–100 μg/mL TiO_2_. Cells were immunofluorescently labeled for AJC proteins ZO-1, E-cadherin, β-catenin, and occludin (*C*). Permeability of the barrier was examined by TEER 96 h after exposure to TiO_2_-NP, and changes were normalized to the time zero for each group (*D*). Scale bar = 20 μm. Data are presented as means ± SE; *n* ≥ 3, *P < 0.05, **P < 0.01.

Titanium dioxide (TiO_2_) is the most abundantly produced nanomaterial in commercial applications and has been shown to be a suitable model to study the adverse pulmonary effect of nanoparticles (4, 20, 61, 81). Therefore, we used TiO_2_-NP as a model to evaluate the effects of environmental particulate matter on the integrity of a model airway epithelial barrier. 16HBE monolayers were exposed to different concentrations of TiO_2_-NP (10, 25, 50, 75, and 100 µg/mL) for 48 h. To dissect the mechanism underlying TiO_2_-NP-induced disruption of the airway epithelial barrier, we sought to examine integrity of the AJC. Specifically, we investigated the effects of TiO_2_-NP exposure on the structure of junctional complexes by immunolabeling protein markers of TJs (ZO-1, occludin) and AJs (E-cadherin, β-catenin). Control cell monolayers displayed a typical “chicken wire” labeling pattern for all tested junctional proteins, which is characteristic of intact TJ and AJ (Fig. 2*A*). This pattern was disrupted in TiO_2_-NP-treated cell monolayers, resulting in either weakened labeling intensity or appearance of gaps and broken strands. Barrier integrity was examined by measuring transepithelial electrical resistance (TEER). TiO_2_-NP caused a reduction in TEER (Fig. 2*B*), which is indicative of the increased ionic permeability of epithelial monolayers. Similar TiO_2_-NP-induced AJC disruption was observed in human primary airway epithelial cell monolayers (Fig. 2, *C* and *D*). Based on these results, 50 μg/mL TiO_2_-NP was chosen for subsequent experiments, as this dose induced significant AJC disruption and permeability, without apparent toxicity. Overall, these results suggest that TiO_2_-NP exposure disrupts the integrity of a model airway epithelial barrier by triggering focal AJC disassembly.

#### TiO_2_-NP enhances the effects of RSV in disrupting a model airway epithelial barrier.

**Fig. 3.**
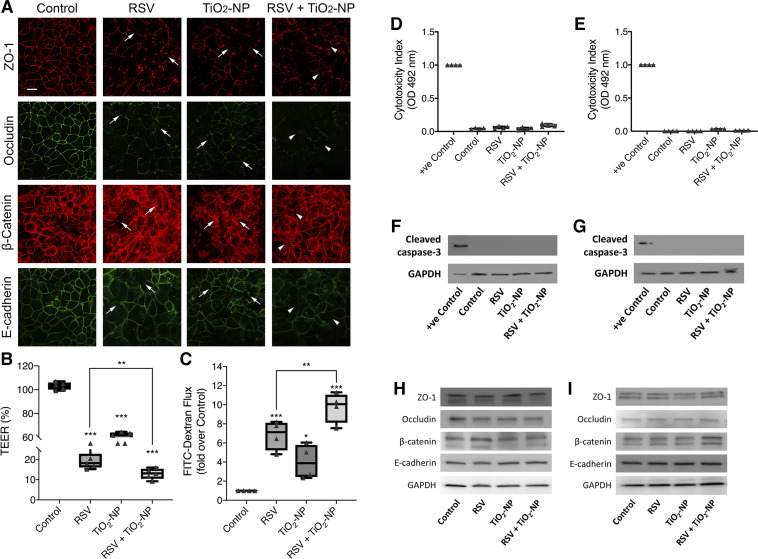
Nanoparticles compound respiratory syncytial virus (RSV)-induced apical junctional complex (AJC) disruption in vitro. Epithelial cells were exposed to RSV (MOI = 0.5) and/or titanium dioxide nanoparticles (TiO_2_-NP; 50 μg/mL). AJC proteins zonula occludens-1 (ZO-1), occludin, β-catenin, and E-cadherin were immunofluorescently labeled (*A*). Arrows indicate disrupted AJC induced by RSV or TiO_2_-NP; arrowheads indicate potentiated disruption. Permeability of the barrier was examined by transepithelial electrical resistance (TEER) at 48 h, and changes were normalized to the time zero for each group (*B*) and FITC-dextran flux assay, for which changes were normalized to the untreated control group (*C*). 16HBE or human primary epithelial cells were apically exposed to TiO_2_-NP for 48 or 96 h, respectively. Lactate dehydrogenase (LDH) assay and Western blot analysis of cleaved caspase 3 in 16HBE (*D* and *F*) and in human primary epithelial cells (*E*–*G*) were performed using supernatants and cell lysates, respectively. 16HBE (*H*) and human primary epithelial cells (*I*) were exposed to TiO_2_-NP and/or RSV. Cell lysates were collected and expression of AJC proteins ZO-1, occludin, β-catenin, and E-cadherin was analyzed by Western blotting; *n* = 2. Densitometry using ImageJ revealed no significant differences among the experimental groups (data not shown). Scale bar = 20 μm. Data are presented as means ± SE; *n* = 3, *P < 0.05, ***P < 0.001 vs. untreated control as determined.

The aforementioned observations, together with published studies highlighting leakiness of the airway induced during RSV infection (98, 101, 117), prompted us to examine whether TiO_2_-NP exacerbates RSV-induced barrier disruption and AJC disassembly. To answer this question, we compared responses of 16HBE cell monolayers to either TiO_2_-NP (50 μg/mL), RSV infection (MOI 0.5), or both in combination. The concentration of TiO_2_-NP was chosen based on the results presented in Fig. 2, and the MOI for RSV infection was established in our previous studies (98, 101). Immunofluorescent labeling demonstrated that coexposure of 16HBE cells to TiO_2_-NP and RSV causes more disruption of AJ and TJ integrity compared with either treatment alone (Fig. 3*A*). Exposure to TiO_2_-NP also caused a reduction in TEER, which was further decreased upon coexposure (Fig. 3*B*). We also observed that TiO_2_-NP exposure caused further increase in transepithelial FITC-dextran passage, suggesting increased monolayer permeability to large molecules (Fig. 3*C*). Importantly, the barrier disruption induced by TiO_2_-NP/RSV exposure was not due to increased cell necrosis or apoptosis in either 16HBE or primary cells, as indicated by LDH-release and caspase-3 cleavage assays (Fig. 3, *D*–*G*). Furthermore, this effect was not due to changes in expression of key AJ and TJ proteins (Fig. 3, *H* and *I*). Quantification by densitometry revealed no significant differences among the experimental groups (data not shown).

#### Nanoparticles increase RSV infection of epithelial cells.

**Fig. 4.**
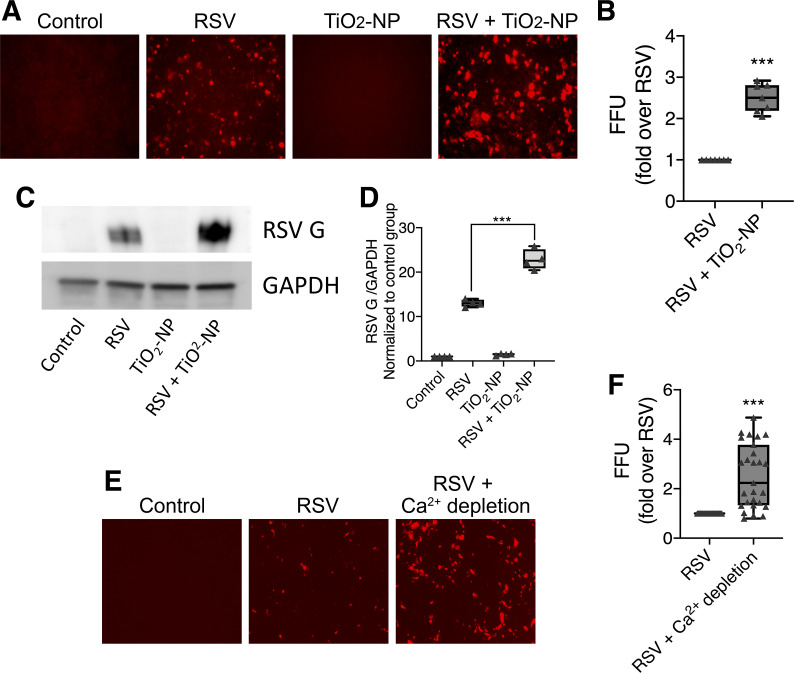
Exposure to titanium dioxide nanoparticles (TiO_2_-NP) potentiates epithelial cell susceptibility to respiratory syncytial virus (RSV) infection. 16HBE cells were incubated with RSV and/or TiO_2_-NP. After 48 h, fluorescent units visible upon expression of the red fluorescent protein after viral replication were quantitatively analyzed by fluorescent-focused unit (FFU) assay (*A* and *B*) and expression of RSV G protein was analyzed by Western blotting. Representative immunoblot images (*C*) and densitometric quantification of protein band intensities normalized to GAPDH and control group are shown (*D*). Cells were incubated overnight with calcium-depleted medium (with or without RSV) and viral replication was quantified by FFU assay (*E* and *F*). Data are presented as means ± SE; *n* = 3, ***P < 0.001 vs. RSV alone.

Next, we sought to investigate whether TiO_2_-NP enhances RSV infection within epithelial monolayers. To test this, 16HBE cells were infected with rrRSV [an engineered virus expressing a red fluorescent protein (RFP) upon replication] with and without TiO_2_-NP. At 48 h, RFP-positive (infected) cells were visualized by fluorescent microscopy and counted (Fig. 4, *A* and *B*). As an alternative assessment of viral infectivity, RSV G protein expression was determined by Western blotting (Fig. 4*C*) and densitometric quantification of protein band intensities normalized to GAPDH and control untreated group (Fig. 4*D*). Both experiments demonstrated that coexposure of airway epithelial cells to both RSV and TiO_2_-NP resulted in increased viral infection compared with cells exposed to RSV alone.

We then examined how TiO_2_-NP exposure may promote RSV propagation within an epithelial monolayer. One possible mechanism is that disruption of epithelial junctions by nanoparticles promotes RSV entrance into the cell. To test this possibility, we compared RSV infection in monolayers in which cells were incubated in a culture medium with a very low concentration (5 μM) of calcium. This low-calcium concentration method has been commonly used to induce epithelial junctional disassembly without causing cell death or detachment from the substrate (32, 116). Fluorescence microscopy revealed ~2.4-fold increase in the number of RSV-infected cells in low-calcium medium compared with cells infected under normal calcium levels (Fig. 4, *E* and *F*). The effects of TiO2-NP and calcium depletion on RSV-infected 16HBE cells were similar, with an equally increased number of fluorescent cells seen in each. Given the resemblance, there is the suggestion of parallel processes occurring with TiO2-NP and low-calcium concentration exposure. Together these results suggest that TiO_2_-NP exposure increases RSV infection by triggering junction disassembly.

#### Nanoparticle-induced disruption of the epithelial barrier is mediated by production of reactive oxygen species.

**Fig. 5.**
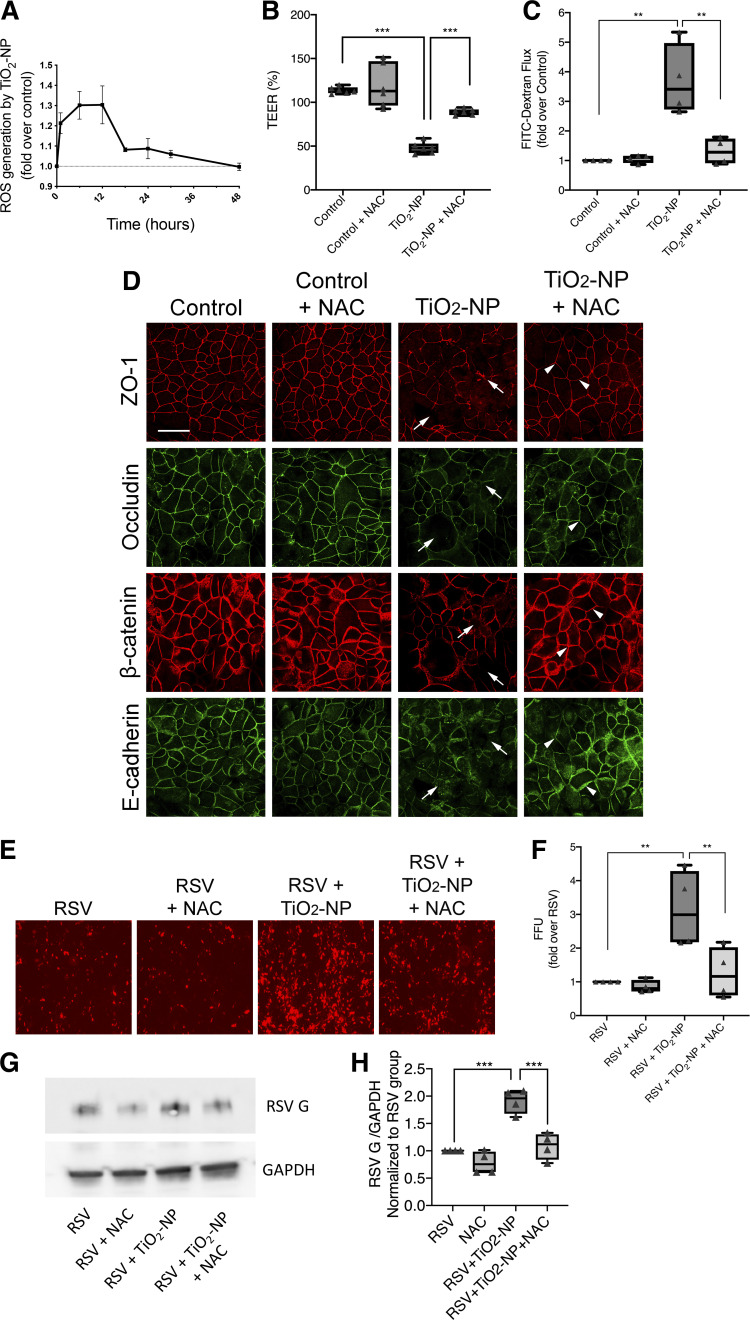
Antioxidants reverse barrier dysfunction induced by titanium dioxide nanoparticles (TiO_2_-NP). Reactive oxygen species (ROS) production was measured by DCFDA assay after exposure to TiO_2_-NP (*A*); 16HBE cells were exposed to TiO_2_-NP and/or antioxidant, *N*-acetylcysteine (NAC) (10 mM), and barrier permeability was examined by transepithelial electrical resistance (TEER). Changes were normalized to the time zero for each group (*B*), FITC-dextran flux assay, with changes normalized to the untreated control group (*C*), and immunofluorescent labeling of apical junctional complex (AJC) proteins zonula occludens-1 (ZO-1), occludin, β-catenin, and E-cadherin (*D*). Arrows indicate disrupted AJC induced by TiO_2_-NP; arrowheads indicate attenuated disruption by NAC. Respiratory syncytial virus (RSV) infection was visualized by fluorescence microscopy and quantitatively analyzed by fluorescent-focused unit (FFU) assay (*E* and *F*). Representative immunoblot images (*G*) and densitometric quantification of protein band intensities normalized to GAPDH and RSV group are shown (*H*). Scale bar = 20 μm. Data are presented as means ± SE; *n* = 3, **P < 0.01, ***P < 0.001 vs. control or TiO_2_-NP groups.

The induction of oxidative stress by TiO_2_-NP has previously been shown (11). Here, we sought to investigate the role of oxidative stress as a molecular mechanism that underlines TiO_2_-NP-induced disruption of the airway epithelial barrier. We probed the involvement of oxidative stress with two experimental approaches: quantification of reactive oxygen species (ROS) production and application of chemical ROS scavenger, *N*-acetylcysteine (NAC). Consistent with previously published data, we found exposure of cells to TiO_2_-NP resulted in a rapid (within 1 h) and sustained (up to 36 h) increase in intracellular ROS (Fig. 5*A*). Furthermore, treatment of TiO_2_-NP-exposed epithelial cell monolayers with well-studied ROS scavenger, NAC, prevented TiO_2_-NP-induced increase in paracellular permeability (Fig. 5, *B* and *C*). Importantly, NAC treatment also attenuated AJC disassembly (Fig. 5*D*) and reversed enhanced RSV infection in TiO_2_-NP-treated cell monolayers (Fig. 5, *E*–*H*). Together these results highlight TiO_2_-NP-dependent ROS generation as an important mechanism for epithelial barrier disruption and increased viral infection in model airway epithelial cell monolayers.

#### Secretion of cytokines is enhanced by TiO_2_-NP.

**Fig. 6.**
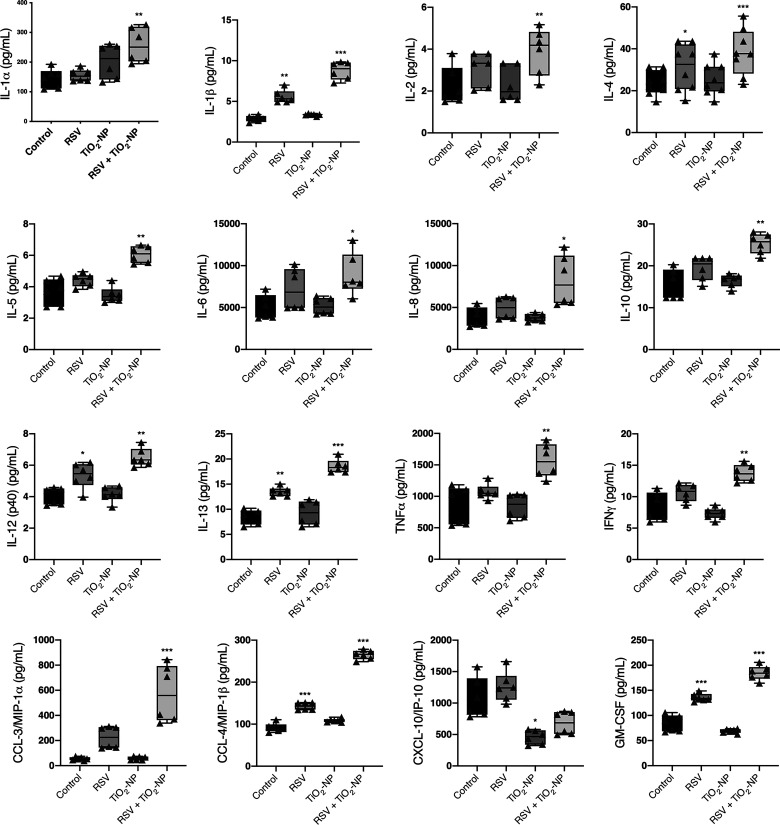
Exposure to titanium dioxide nanoparticles (TiO_2_-NP) modifies proinflammatory cytokine expression during respiratory syncytial virus (RSV) infection. Cytokine concentrations of apical supernatants of 16HBE cells exposed to rrRSV at MOI of 0.5, TiO_2_-NP, or a combination were determined. Cytokines were measured using the Luminex/Magpix magnetic bead platform. Interleukin IL-1α, IL-1β, IL-2, IL-4, IL-5, IL-6, IL-8, IL-10, IL-12 (p40), IL-13, tumor necrosis factor-α (TNFα), interferon-γ (IFNγ), CCL-3 [macrophage-inflammatory protein (MIP)-1α], CCL-4 (MIP-1β), and granulocyte-macrophage colony-stimulating factor (GM-CSF) protein concentrations were higher in supernatant of cells exposed to both TiO_2_-NP and RSV. CXCL-10 [IFNγ-induced protein 10 (IP-10)] expression was reduced after exposure to TiO_2_-NP. Data are presented as means ± SE; *P < 0.05, **P < 0.01, ***P < 0.001 vs. control group, *n* = 3.

We evaluated cytokine responses of epithelial cell monolayers during exposure to TiO_2_-NP, RSV, or a combination. Supernatants were collected after 48 h of exposure and were analyzed by Magpix Luminex. The response was characterized by further increased release of multiple proinflammatory cytokines and chemokines into epithelial cell supernatant (Fig. 6). Cytokines that were not altered include interleukins IL-1ra, IL-3, IL-7, IL-12 (p70), IL-15, IL-17, IFNα2, TNFβ, CCL-2 [monocyte chemoattractant protein (MCP)-1], CCL-5 (RANTES), CCL-11 (eotaxin), epidermal growth factor (EGF), granulocyte-colony stimulating factor (G-CSF), and vascular endothelial growth factor (VEGF).

#### Nanoparticles induce airway inflammation and exaggerate RSV-induced inflammatory response in vivo.

**Fig. 7.**
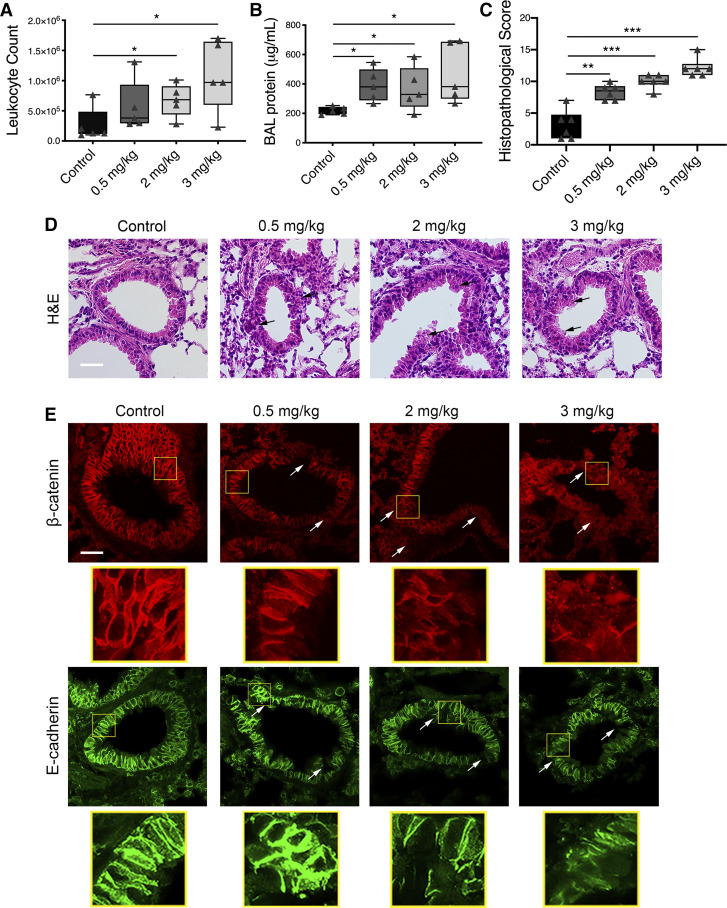
Exposure to titanium dioxide nanoparticles (TiO_2_-NP) causes dose-dependent inflammation. Mice were intranasally inoculated with TiO_2_-NP (0.5–3 mg/kg). On *day 4*, lungs and bronchoalveolar lavage (BAL) were harvested and analyzed for white blood cell (*A*), protein (*B*), and histopathological score and photomicrographs of hematoxylin-eosin (H&E)-stained lung tissue section (*C* and *D*), and disruption of apical junctional complex (AJC) proteins β-catenin and E-cadherin (*E*). Arrows point to areas of peribronchial inflammation (black) and AJC disruption (white) after exposure to TiO_2_-NP. Scale bar = 40 μm. The panels below each image show magnified junctional protein. Data are presented as means ± SE; *n* = 5 mice per group of 2 independent experiments, *P < 0.05 vs. control group, **P < 0.01, ***P < 0.001 vs. control group.

A series of experiments was designed to test the pathophysiological relevance of our in vitro findings. To examine the effects of nanoparticles on the airway epithelial barrier in vivo, 6- to 8-wk-old mice were intranasally exposed to different doses of TiO_2_-NP (0.5–3 mg/kg body weight) or control as described in materials and methods. Mice were euthanized on *day 4* postinoculation to harvest their lung tissue and collect bronchoalveolar lavage (BAL). BAL was used to quantify infiltrating blood cells, whereas harvested lung sections were subjected to either histochemical or immunofluorescence labeling analysis. TiO_2_-NP inoculation caused dose-dependent leukocyte infiltration into the airways (Fig. 7*A*), suggesting airway inflammation. The total protein concentration was significantly increased in BAL of TiO_2_-NP-exposed animals compared with control mice on *day 4* postinoculation (Fig. 7*B*), suggesting increased airway permeability, as there is a flux of large molecules across the pulmonary barrier. Furthermore, hematoxylin and eosin (H&E) staining of lung sections demonstrated that TiO_2_-NP administration caused a dose-dependent inflammatory response, manifested by airway wall thickening and immune cell accumulation in the peribronchial spaces (Fig. 7, *C* and *D*). Finally, immunolabeling revealed disruption of β-catenin and E-cadherin integrity in TiO_2_-NP-exposed airways in vivo, suggesting disarrangement of the airway barrier (Fig. 7*E*).

Given our findings, we next sought to elucidate whether TiO_2_-NP can also exacerbate RSV-induced airway inflammation in vivo. Animals were divided into four experimental groups and inoculated intranasally with either TiO_2_-NP (2 mg/kg), RSV (9.6 × 10^6^ PFU) (117), RSV and TiO_2_-NP in combination, or vehicle (PBS). The animals were euthanized 4 days after inoculation.

**Fig. 8.**
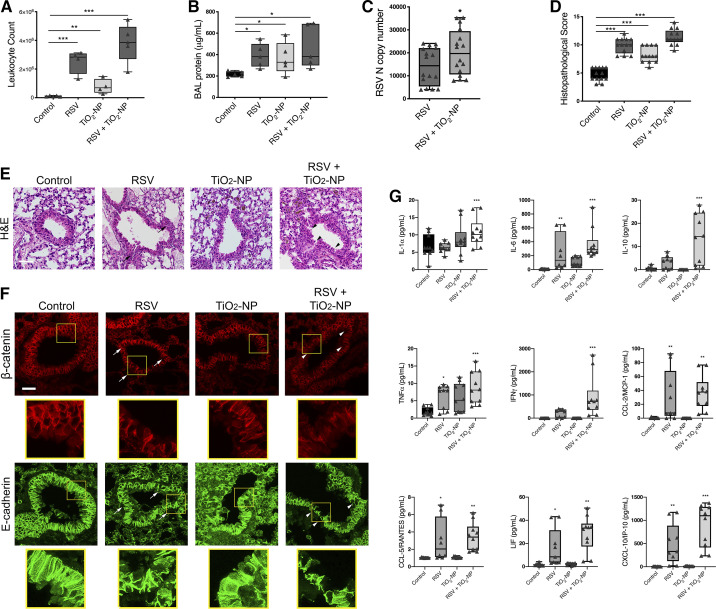
Exposure to titanium dioxide nanoparticles (TiO_2_-NP) enhances respiratory syncytial virus (RSV)-induced barrier disruption in vivo. Mice were intranasally inoculated with TiO_2_-NP (2 mg/kg), followed immediately by RSV. On *day 4*, lungs and bronchoalveolar lavage (BAL) were harvested and analyzed for white blood cell infiltration (*A*), protein (*B*), RSV nucleocapsid (N) copy number in lung homogenates (*C*), and histopathological score and photomicrographs of hematoxylin-eosin (H&E)-stained lung tissue section (*D* and *E*), and disruption of apical junctional complex (AJC) proteins β-catenin and E-cadherin (*F*). Arrows point to areas of peribronchial inflammation (black) and AJC disruption (white) after exposure to RSV or TiO_2_-NP; arrowheads point to areas of exaggerated inflammation (black) and AJC disruption (white) after coexposure. Scale bar = 40 μm. Data are presented as means ± SE; *n* = 10 mice per group, *P < 0.05, **P < 0.01, and ***P < 0.001 vs. control or TiO_2_-NP groups. Exposure to TiO_2_-NP modifies proinflammatory cytokine expression during RSV infection in vivo (*G*). Cytokine concentrations of BAL of mice exposed to rrRSV, TiO_2_-NP, or a combination were determined. Interleukin (IL)-1α, IL-6, IL-10, tumor necrosis factor-α (TNFα), interferon-γ (IFNγ), monocyte chemoattractant protein (MCP)-1, RANTES, LIF, and IFNγ-induced protein 10 (IP-10) protein concentrations were higher in BAL fluid of mice exposed to both TiO_2_-NP and RSV. Data are presented as means ± SE; *n* = 5 mice per group, of 2 independent experiments, *P < 0.05, **P < 0.01, ***P < 0.001 vs. control group.

White blood cell quantification and BAL total protein concentrations of all experimental groups were significantly increased in BAL compared with control mice. These parameters were higher in mice that were exposed to both RSV and TiO_2_-NP compared with RSV infection alone (Fig. 8, *A* and *B*). A quantitative RT-PCR analysis found increased mRNA expression of a specific viral N gene in lung homogenates of mice inoculated with both RSV and TiO_2_-NP in comparison to mice infected with RSV alone (Fig. 8*C*). H and E staining of harvested lung tissue and histopathological scoring demonstrated a greater inflammatory response after coexposure to RSV and TiO_2_-NP compared with RSV infection alone (Fig. 8, *D* and *E*). Likewise, immunolabeling and confocal microscopy demonstrated that TiO_2_-NP exposure exaggerates RSV-induced disruption of airway epithelial junction proteins β-catenin and E-cadherin in vivo (Fig. 8*F*). Finally, cell-free BAL from mice exposed to PBS control, 2 mg/kg TiO_2_-NP, RSV, or a combination thereof was collected on *day 4*. Samples were analyzed by Magpix Luminex (Fig. 8*G*). Levels of proinflammatory cytokines IL-1α, IL-6, IL-10, TNFα, IFNγ, CCL-2 (MCP-1), CCL-5 (RANTES), leukemia inhibitory factor (LIF), and CXCL-10 (IP-10) increased upon coexposure. Cytokines that remained unchanged were interleukins IL1β, IL-2, IL-3, IL-4, IL-5, IL-7, IL-9, IL-12 (p40 and p70), IL-13, IL-15, IL-17, CXCL-1 [keratinocyte chemoattractant (KC)], CXCL5 (LIX), CCL-3 [macrophage-inflammatory protein (MIP)-1α], CCL-4 (MIP-1β), CXCL-9 [monokine induced by IFNγ (MIG)], CXCL-2 (MIP-2), CCL-11(eotaxin), G-CSF, GM-CSF, macrophage-colony stimulating factor (M-CSF), and VEGF.

Together, these data suggest that RSV enhances the effects of TiO_2_-NP by compounding epithelial AJC disruption and inducing higher airway inflammation.

## DISCUSSION

The data here describe how nanoparticles enhance RSV-induced airway epithelial cell barrier dysfunction and highlight the potential role of environmental exposures in the pathogenesis of respiratory disease. As the first line of defense against inhaled pathogens, the epithelium is frequently exposed to pollutants and particulate matter. Multiple epidemiologic studies have linked pollution exposure to pulmonary diseases, such as asthma, bronchitis, otitis media, and severe viral infection (4, 6, 10, 12, 29, 37, 58, 70, 80a, 92, 108). In addition, a recent, large-scale epidemiological study demonstrates that in utero exposure to ultrafine particles increases childhood asthma incidence (131). Understanding the mechanisms by which particulate matter exposure contributes to airway disease is vital.

We and others have previously demonstrated that RSV stimulates disassembly of epithelial AJC, resulting in increased permeability and a “leaky airway” both in vitro and in vivo (35, 99, 117). We have previously shown that AJC disassembly is essential for an RSV-dependent increase in permeability of the murine epithelial barrier. Dysfunction of AJC may perpetuate airway inflammation by facilitating “outside/in” translocation of inhaled particles, allergens, and pathogens. We found that nanoparticles augment the effects of RSV by furthering disruption of epithelial AJC structure and increasing permeability. These effects were echoed in vivo. We previously demonstrated that ZO-1 levels were decreased, occludin became mislocalized, and claudin-2 levels increased following RSV infection in a murine model (117). Mice given a single intranasal dose of TiO_2_-NP demonstrated exaggerated inflammation of the airways and enhanced disruption of AJ protein, β-catenin, and epithelial marker, E-cadherin, architecture compared with those given RSV alone.

After demonstrating that TiO_2_-NP heightened the inflammatory response in both RSV-infected epithelial monolayers and mice, further investigation was warranted to determine the mechanisms in which this was occurring. This response was characterized by further increased release of multiple proinflammatory cytokines and chemokines into both epithelial cell supernatant and mouse BAL. The antiviral response to RSV infection has been associated with increases of various cytokines and chemokines (43, 105, 123), some of which have been shown to be associated with more severe disease. For instance, GM-CSF levels are higher in infants with RSV infection requiring ventilation (24), and the level of CCL-3 (MIP-1α) has a positive correlation with the duration of required supplemental oxygen (33). MIP-1α is also shown to be present in the lower airway during severe RSV infection (43). Similarly, increased levels of IL-3 and IL-12 (p40) during RSV infection are correlated with subsequent development of recurrent wheeze (9), and increased VEGF, G-CSF, and IL-10 levels have been shown to increase the risk of post-virus-induced wheeze, persisting after the RSV episode (91). In addition, studies have indicated that cytokines including IL-4 and IL-13 have a disruptive effect on airway epithelial barrier function (107). In contrast to the other cytokines, the level of CXCL10 was suppressed in both TiO_2_-NP exposed alone and coexposure of TiO_2_-NP and RSV. CXCL10 is a chemokine involved in the recruitment of Th1 type immunity, which involves cell-mediated adaptive immune responses and antiviral activity. Our findings are similar to a recent study that showed reduction of CXCL10 from airway epithelial cells exposed to diesel exhaust particulate (77). However, some in vivo experiments showed that TiO_2_ did not change the level of CXCL10 (86), but longer exposure of repeated doses of TiO_2_ increased the CXCL10 level (4). Overall these data provide new information on how coexposure with TiO_2_ changes the inflammatory response to RSV. It is of great importance to explore the chronic exposure to TiO_2_ and RSV infection on alteration of the airway barrier and airway inflammation, which is beyond the scope of the current study.

In addition to increased cytokine production, we demonstrate that TiO_2_-NP causes a steady, time-dependent elevation in ROS generation that is sustained over 48 h. The adverse effects of NP have been widely recognized as a result of reactive oxygen species-induced airway inflammation (5, 44, 55, 59, 69, 72, 84). In addition to disrupting tight junctions (60, 96), evidence suggests that free radicals induce pulmonary inflammation (19, 67), increase airway permeability, destabilize actin (13, 54), and enhance severity of viral infection (66). *N*-acetylcysteine (NAC), a radical scavenger, has been widely used as a research tool for investigating the role of ROS (3, 119). After administering NAC, we saw a decrease in TiO_2_-NP-induced AJC disruption. Exposure to NAC also attenuated increases in permeability. Given this, TiO_2_-NP exposure exacerbates RSV-induced AJC disruption through a heightened inflammatory response consisting of increased ROS and cytokine production.

We also observed an increase in RSV infection after exposure to nanoparticles, but the exact molecular mechanisms responsible remain unclear. There is growing literature pointing to the barrier disruption as a means of facilitating viral dissemination (25, 36, 111, 120, 125). Disruption of TJ proteins has been shown to enhance infection of norovirus (121) and facilitate access of human rhinovirus (136), RSV (73), and rotavirus (82) to viral receptors in various organs. Degradation of TJ has also been shown to enhance viral and bacterial dissemination (1, 26, 71, 124, 127, 135). Consistent with this phenomenon, selective, artificial disruption of epithelial AJC by calcium depletion revealed a substantial increase in viral infection, suggesting that TiO_2_-NP mediates infection by triggering junction disassembly. Interestingly, exposure to NAC attenuated the enhanced permissiveness to infection, again confirming the role of ROS in both AJC disruption and viral infection. Conversely, it is likely that other mechanisms contribute to this phenomenon; for example, previous studies have shown that permissiveness to RSV may be increased by nerve growth factor-induced autophagy induced by TiO_2_-NP (20) or disrupted mitochondrial metabolism after exposure to cadmium (49). Ozone exposure was shown to induce the cleavage of influenza A virus membrane proteins, leading to increased susceptibility (57).

In this study, we chose to study TiO_2_-NP to characterize a model of physiological exposure, as it is the most abundantly produced nanomaterial in commercial applications with demonstrated potential for causing inflammation and injury of the lower airways. In addition, multiple studies have shown that TiO_2_-NP is a suitable model to study the pulmonary adverse effects of NP (4, 20, 61, 81). However, TiO_2_-NP may not fully represent generalized exposure to pollution or nanoparticulate matter. For the current study we have chosen not to study ambient pollutants, such as diesel exhaust particles (DEP), because DEP has a number of physical parameters that cannot be easily controlled, whereas the TiO_2_-NP in these experiments have been engineered with specific size, shape, and surface characteristics. Also, DEP carry large quantities of adsorbed organic chemicals on a per-mass basis, such as carbon core-carrying trace metals, sulfates, and ammonium that may exhibit other toxic effects out of the scope of current application.

It is plausible that reduced barrier integrity as a consequence of NP exposure may increase allergen uptake and exacerbate the subsequent immune responses (35, 46, 63, 99). Numerous studies have linked disrupted AJC to exacerbation of airway disease (35). For example, studies have shown that biopsies and brushings from patients with moderate and severe asthma demonstrate reduced barrier function and reduced junctional protein expression, particularly of ZO-1 and E-cadherin (27, 133). In addition, the role of the airway epithelial barrier in regulating the pathogenesis of asthma has been previously investigated (45), and, recently, ultrafine particle exposure has been positively associated with childhood asthma incidence (64).

Taken together, our findings indicate that exposure to TiO_2_ nanoparticles exacerbate RSV-induced AJC disruption. The strength of our study is in showing the mechanism in vitro with evidence of similar features in vivo.

There is a need for further in vitro studies and animal models to elucidate the interaction between NP exposure and viral infection especially during childhood, a critical window of vulnerability to environmental stimuli. The observations described in this paper may enable interventional strategies to increase resistance of the airways against airborne biological, physical, and chemical insults, particularly the pathogenic effects of RSV infection. Future studies will need to determine whether differences in NP exposure can explain why some children experience more severe airway disease with RSV infection.

## GRANTS

This work was supported by the Mark Lauer Pediatric Research Grant, Cleveland Clinic Children’s (CCS/FR), as well as National Institutes of Health National Institute of Allergy and Infectious Diseases Grant K08-AI-112781 (F.R.), National Heart, Lung, and Blood Institute Grants R01-HL-148057 (F.R.), and R01-HL-061007 (G.P.). This work also utilized the Leica SP8 confocal microscope that was purchased with funding from NIH Shared Instrument Grant (SIG) S10-OD019972.

## DISCLOSURES

No conflicts of interest, financial or otherwise, are declared by the authors. The authors have no financial relationship with a biotechnology and/or pharmaceutical manufacturer that has an interest in the subject matter or materials discussed in the submitted manuscript

## AUTHOR CONTRIBUTIONS

F.R. conceived and designed research; C.C.S., T.J.H., S.L., V.B., and F.R. performed experiments; C.C.S., D.T.L., G.P., and F.R. analyzed data; C.C.S., T.J.H., G.P., and F.R. interpreted results of experiments; C.C.S. and F.R. prepared figures; C.C.S., D.T.L., and F.R. edited and revised manuscript; C.C.S., T.J.H., D.T.L., S.L., V.B., G.P., and F.R. approved final version of manuscript.
